# Regulator of G-Protein Signaling 18 Controls Both Platelet Generation and Function

**DOI:** 10.1371/journal.pone.0113215

**Published:** 2014-11-18

**Authors:** Nathalie Delesque-Touchard, Caroline Pendaries, Cécile Volle-Challier, Laurence Millet, Véronique Salel, Caroline Hervé, Anne-Marie Pflieger, Laurence Berthou-Soulie, Catherine Prades, Tania Sorg, Jean-Marc Herbert, Pierre Savi, Françoise Bono

**Affiliations:** 1 Early to Candidate (E2C), Sanofi, Toulouse, France; 2 SCP Biologics, Sanofi, Vitry-Sur-Seine, France; 3 Department of Scientific Operations PhenoPro, Mouse Clinical Institute (MCI), Strasbourg, France; Royal College of Surgeons, Ireland

## Abstract

RGS18 is a myeloerythroid lineage-specific regulator of G-protein signaling, highly expressed in megakaryocytes (MKs) and platelets. In the present study, we describe the first generation of a RGS18 knockout mouse model (RGS18-/-). Interesting phenotypic differences between RGS18-/- and wild-type (WT) mice were identified, and show that RGS18 plays a significant role in both platelet generation and function. RGS18 deficiency produced a gain of function phenotype in platelets. In resting platelets, the level of CD62P expression was increased in RGS18-/- mice. This increase correlated with a higher level of plasmatic serotonin concentration. RGS18-/- platelets displayed a higher sensitivity to activation *in vitro*. RGS18 deficiency markedly increased thrombus formation *in vivo*. In addition, RGS18-/- mice presented a mild thrombocytopenia, accompanied with a marked deficit in MK number in the bone marrow. Analysis of MK maturation *in vitro* and *in vivo* revealed a defective megakaryopoiesis in RGS18-/- mice, with a lower bone marrow content of only the most committed MK precursors. Finally, RGS18 deficiency was correlated to a defect of platelet recovery *in vivo* under acute conditions of thrombocytopenia. Thus, we highlight a role for RGS18 in platelet generation and function, and provide additional insights into the physiology of RGS18.

## Introduction

From the bone-marrow, where megakaryopoiesis and thrombopoiesis occur (i.e. proliferation, differentiation, migration, maturation of megakaryocytes and proplatelets into platelets) to injured tissue, where platelets get activated, there are many signaling pathways largely dependent on G protein-coupled receptors (GPCRs) involved. GPCRs are by far the most extensively validated class of therapeutic targets. Over half of the existing drugs currently on the market are GPCR ligands. However, only a small fraction of these receptors are targeted by drugs [1], [2]. Drug discovery efforts for GPCR ligands have traditionally focused on targeting the orthosteric binding site of the receptors. One of the key issues in this regard is that the orthosteric binding sites across members of a single GPCR subfamily are often highly conserved, making it difficult to achieve high selectivity for specific GPCR subtypes. During the last decade, the idea of targeting allosteric sites – so called allo-targeting - as a novel approach to GPCR drug discovery has become a predominant approach [3]. Rather than targeting receptors directly, one could modulate signaling cascades downstream of receptor activation.

It is now well recognized that the regulators of G protein signaling (RGS) play essential roles in GPCR signaling [4]. RGS proteins are a family of cellular proteins with a conserved RGS domain (also called RGS-box) of about 120 amino-acid residues. RGS proteins specifically interact with the α subunits of G proteins, greatly enhance the intrinsic GTPase activities of Gα, and accelerate the hydrolysis of GTP to GDP by Gα, thus converting G proteins from a GTP-bound active state to a GDP-bound inactive state and terminating G protein-mediated signaling [5]. There are over 20 members in the mammalian RGS family. Based on sequence similarities and features of structural domains, they have been classified into 9 subfamilies [6]. *In vitro* and *in vivo* studies have provided strong evidence supporting that RGS proteins display remarkable specificity and selectivity in their regulation of GPCR-mediated physiological events [7]. The spatiotemporal-specific expression of RGS proteins and their target components, as well as the specific protein-protein recognition and interaction through their characteristic structural domains and functional motifs, are continually emerging as determinants for RGS selectivity and specificity. Recent research data are converging to highlight RGS proteins as attractive targets for the development of potential future therapeutics [8], [9]. An RGS inhibitor would be expected to enhance GPCR signaling, and should do so in a tissue- or pathway-specific manner. Modulating RGS activity would then be a useful therapeutic strategy to control GPCR signaling in a unique way.

In 2001, several groups independently identified RGS18, and all agreed that RGS18 expression appears to be relatively restricted to bone marrow-derived cells [10]–[12]. RGS18 was found abundantly and predominantly expressed in platelets and, to a less extent in megakaryocytes (MKs) and leukocytes, but not in erythrocytes. In addition to RGS18, human MKs also highly express RGS16 [12], [13]. RGS16 and RGS18 have their respective transcripts upregulated during MK differentiation and RGS16 was shown to act as a negative regulator of CXCR4 signaling in MKs [13]. However, in contrast to the restricted expression profile of RGS18, RGS16 is widely expressed. The expression patterns of RGS transcripts have been evaluated in platelets, and RGS18 was found to have the highest expression level, followed by RGS6, RGS10, and RGS16 [14]–[16]. The functional significance of RGS proteins in platelets has recently been validated in a mouse model expressing a serine to glycine substitution at position 184 in the α subunit of Gi2, the G protein that couples to platelet P2Y12 receptors for ADP [17]. The mutant Gαi2 was shown to be unable to interact with RGS, and loss of Gαi2: RGS interactions produced a gain of function in platelet activation. Most platelet agonists (thrombin, ADP, TXA2 …) activate members of the GPCR family, making G proteins and RGS proteins logical targets for regulation. On the basis of the abundant and specific expression pattern of RGS18 in platelets and MKs, we hypothesized that it might be a key regulator of GPCR signaling in these cells and to address this question, we have generated and phenotyped mice lacking a functional RGS18 gene.

Here we report that RGS18 deficiency produces a pro-thrombotic phenotype in mice, shifting the dose-response curve for platelet aggregation induced by thrombin to the left *in vitro* and increasing thrombus formation at sites of arteriovenous shunt *in vivo*. Moreover, we also show that RGS18 deficiency is sufficient to prime platelet activation even before an agonist is added. In addition, RGS18-/- mice present a mild thrombocytopenia, accompanied by a reduced number of megakaryocytes (MK) in their bone marrow. Analysis of MK maturation *in vitro* and *in vivo* reveals that RGS18-/- mice display a defective megakaryopoiesis. Finally, RGS18 was shown to display a thrombocytopoietic activity, as revealed by deficient platelet recovery in RGS18-/- mice under acute thrombocytopenia conditions.

## Methods

### Generation of RGS18 mutant mice

RGS18 knockout mice were generated at the MCI (Mouse Clinical Institute/Institut Clinique de la Souris, Strasbourg, France). The targeting vector was constructed as described follows: a 1kb PCR fragment encompassing exon 4 was cloned (PCR done on embryonic stem (ES) cell genomic DNA 129S2/SvPas genetic background) into an MCI proprietary vector, resulting in step 1 plasmid. This MCI vector has a floxed neomycin resistance cassette. Two PCR fragments of 4.2 kb corresponding to the 5′ homologous arm and 3 kb corresponding to the 3′ homologous arm were amplified and cloned successively into the step 1 plasmid to generate the final targeting construct. The linearized construct was electroporated into 129S2/SvPas mouse ES cells. After selection, targeted clones were identified by PCR using external primers and further confirmed by Southern blot with 5′ and 3′ external probes. The positive ES clones were injected into C57BL/6J blastocysts, and male derived chimeras gave germline transmission. First L3 mice were successively bred with Flp and Cre transgenic mice (on C57BL/6 genetic background) to generate heterozygous mice with a deleted neomycin cassette and an inactivated RGS18 locus. Mice were then backcrossed for 4 consecutive generations to C57BL/6J genetic background. Deletion of exon 4 was confirmed at the DNA and transcript level.

### Animals

C57BL/6J mice (Charles River Laboratories, Wilmington, MA, USA) weighing between 27 to 34 g were used throughout the study. The animals were kept on standard diet *ad libitum* and had free access to tap water. For the length of the experiment, the animals were maintained at an external temperature of 21°C +/−10%. The housing air was changed 15 to 20 times per hour. The animals were used after one week of quarantine. This protocol was approved by the "Comité d'Ethique Pour les Animaux de Laboratoire" (Animal Care and Use Committee) of Sanofi R&D.

### *In silico* analysis of RGS18 promoter

The RGS18 promoter sequence (from −1000 bps to +100 bps) of human, mouse, rat, cow and dog were taken from Ensembl. Sequences were aligned and analyzed for phylogenetic conserved transcription factor binding sites (TFBS) using MatInspector and frameWorker programs from Genomatix software suite.

### Peripheral blood cell counting

Mice were anesthetized by using isoflurane gas (Aerrane; Baxter, Lessines, Belgium). Peripheral blood samples were obtained from the retro-orbital plexus with 75 mm EDTA capillary tubes (Sarstedt, Montreal, Canada). Complete blood cell counts were performed with the VetABC animal cell counter (Horiba ABX SAS, Montpellier, France).

### von Willebrand factor (vWF) staining on bone marrow sections

Femora were fixed in formalin, decalcified in Dc3 (Labonord, Temple-mars, France) and embedded in paraffin. Sections (5 µm) were deparaffined, rehydrated through serially diluted ethanol solutions to distilled water. After trypsin (Invitrogen, Carlsbad, CA, USA) treatment (10 minutes at 37°C), immunohistochemistry was performed using the Autostainer (Dako, Carpinteria, CA, USA). Endogenous peroxidase activity was quenched with 3% (wt/vol) hydrogen peroxide, and the sections were blocked with 10% (vol/vol) normal goat serum. The von Willebrand factor (Dako) staining (12.4 µg/ml) was carried out with a standard 3-stage immunoperoxidase method using the Vectastain Elite ABC peroxidase kit (Vector Laboratories, Peterborough, UK). Diaminobenzidine was used as chromogen. Counterstaining was performed with hematoxylin. For each section, the surface of the bone marrow was measured with the 4x objective (Nikon Eclipse E800) and the number of positive vWF MKs was counted using the 20x objective. Morpho Expert software (Explora Nova, La Rochelle, France) was used for the quantification.

### Flow cytometric analysis

Blood samples were collected by retro-orbital puncture on hirudin at the final concentration of 20 µg/ml. Cells were incubated with FITC rat anti-mouse CD41 (BD Biosciences, San Jose, CA, USA; 100 ng/µl) or with FITC rat anti-mouse CD42b (Emfret Analytics, Eibelstadt, Germany; diluted to 1∶5) for 10 minutes at room temperature. To measure basal platelet activation, cells were stained with rat anti-mouse P-Selectin (Emfret; diluted to 1∶5) for 10 minutes at 37°C. After staining, cells were fixed with Cellfix (BD Biosciences) according to the manufacturer's instructions, and analyzed with CyanADP cytometer (Dako). MKs and platelets were discriminated according to their forward and side scatter parameters. To quantify splenic MKs and platelets, cells were isolated by homogenization of mice spleen in IMDM medium containing 2% heat-inactivated FBS using the Ribolyzer instrument (Hybaid, Teddington, UK). 100µl of the homogenate were stained and red blood cells were lysed with EasyLyse reagent (Dako) before cytometer analysis.

### *In vitro* colony assays

Mice were lethally injected intraperitoneally with sodium pentobarbital (150 mg/kg). Bone marrow cells were flushed from femora and tibias with IMDM medium containing 2% heat-inactivated FBS. Single-cell suspensions were prepared under sterile conditions and enriched for Lineage-depleted (Lin^-^) cells using a Spin Sep mouse hematopoietic progenitor negative selection kit (StemCell Technologies, Vancouver, Canada). Lin^-^ marrow cells (5.10^4^) were then cultured in double chamber slides with Mega-Cult-C media (StemCell Technologies) in presence of rmIl-3 (Peprotech, Rocky Hill, NJ, USA; 10 ng/ml), rmIl-6 (Peprotech; 3 ng/ml) and rhTPO (Peprotech; 50 ng/ml). After 11 days of culture, the slides were dehydrated and colonies were stained with FITC rat anti-mouse CD41 (BD Biosciences). Slides were scored microscopically, and MK colonies (CFU-MKs) were defined as colonies with at least 3-4MKs.

### Ploidy analysis of CD41^+^ bone marrow megakaryocytes

Bone marrow suspensions were prepared in a phosphate-buffered saline (PBS)-bovine serum albumin (BSA) buffer (PBS containing 0.5% BSA). Cells (5.10^6^) were labeled for 30 min at 4°C with 1.25 µg of a fluorescein isothiocyanate (FITC)-conjugated monoclonal antibody against CD41 (rat anti-mouse glycoprotein IIb; Pharmingen) and gently washed twice in PBS-BSA. The pellet was resuspended in 200 µl of PBS and 4 ml of a cold solution of 70% ethanol in PBS was added. After incubation for 1 h at 4°C, the suspension was centrifuged, the cells were resuspended in 100 µl of PBS and propidium iodide (Sigma-Aldrich, Gillingham, UK; 2 mL [50 µg/mL]) and RNAase (Sigma; 100 µg/ml) in PBS were added for 30 min at 37°C. The ploidy distribution in the CD41+ population was determined by two-color flow cytometry (BD FACSCanto; BD Biosciences).

### Platelet depletion

Mice were given a sterile intraperitoneal injection of anti-mouse Gp1bα (Emfret; 2µg/g). 4 hours later, mice were tailed bled to determine baseline blood cell count. Then, platelet count was measured at 48, 72, 96, and 168 hours post injection by retro-orbital sampling.

Thrombocytopenia was also produced by the intraperitoneal injection of busulfan (Myleran; Burroughs-Wellcome, Research Triangle, NC, USA). Busulfan was injected to mice intraperitoneally at 30 mg/kg (in a 25% polyethylene glycol-400 solution) at day 0. Platelet counts were performed following the same procedure as described earlier at days 7, 11, 15, and 24.

### Platelet Clearance

Mice platelets were biotinylated *in vivo* by infusion with NHS-biotin (Calbiochem, La Jolla, CA, USA). 10 mg NHS-biotin/kg body weight were dissolved in DMSO, then diluted into sterile saline solution at 1 mg/ml. The solution was slowly injected via the lateral tail vein of mice with a 26G1/2 needle. Blood collection was realized by retro-orbital sampling at days 1, 2, 3, 4 and 7 after biotin injection. Samples were stained with FITC rat anti-mouse CD41 (BD Biosciences; 100 ng/µl) and then incubated with PE-Streptavidin (Calbiochem; diluted to 1∶3). The platelet biotinylation rate was analyzed by flow cytometry (CyanADP, FITC staining).

### Platelet-rich Plasma (PRP) Preparation

Venous blood was collected into tubes containing a 3.8% trisodium citrate solution (9∶1 vol/vol). Platelet-rich plasma (PRP) was obtained by centrifuging blood samples at 200×g for 10 min and left for 30 min at 37°C prior to stimulation.

### Serotonin release assay

An aliquot of PRP was centrifuged at 2000×g for 10 min to obtain platelet-free plasma (PFP). 100 µl of PFP were used in the serotonin ELISA kit (Labor Diagnostika Nord Gmbh, Nordhorn, Germany) for the measurement of free (not bound to platelets) serotonin according to the manufacturer's instructions.

### αIIbβ3 Activation

Platelet activation was determined according to the method of Bergmeier [18]. Platelets in PRP were stimulated at 37°C for 3 minutes using ADP (2.5 µM), collagen (10 µg/ml), or increasing doses of TRAP (125 µM-500 µM). Platelets were then stained with JON/A-PE32 (Emfret Analytics, Wuerzburg, Germany) for 10 minutes at room temperature and analyzed by flow cytometry.

### Aggregometry

Platelet aggregation was determined according to the Born method [19] on a dual-channel Chrono-Log aggregometer (Chrono-log, Havertown, PA, USA). The experiment was performed at 37°C under constant stirring conditions (1200 rpm). Increasing concentrations of thrombin (0.01–1 UI/ml) were added to PRP and light transmission was recorded over 6 minutes.

### Silk thread arterio-venous shunt model

Two 6 cm-long polyethylene tubing (0.28 and 0.61 mm inner and outer diameter, respectively) linked to a central part (3 cm-long; 0.58 and 0.96 mm inner and out diameter, respectively) containing a 3 cm-long silk thread and filled with saline solution were placed between the right carotid artery and the left jugular vein in anaesthetized animals (xylazine 20 mg/kg + ketamine 100 mg/kg ip). The central part of the shunt was removed after 10 minutes of blood circulation and the silk thread carrying the thrombus was pulled out. The wet weight of the thrombus was determined.

### Experimental tail transection bleeding model

Mice were anesthetized by intraperitoneal injection of xylazine (20 mg/kg) with ketamine (100 mg/kg). The bleeding time was determined according to Dejana et al. [20] adapted for mice, by transection of the tail 2 mm from the tip. Blood was carefully blotted on a filter paper every 15 sec during the two first minutes and every 30 sec thereafter. The observation period was limited to 45 minutes. Hemostasis was considered to be achieved when no more bloodstaining was observed over 1 min. Raw data correspond to the bleeding time in minutes.

### Statistical analysis

Results are presented as means ± SD. The data were analyzed by using a 1-way analysis of variance (ANOVA) with Student *t* test or Dunnett test. *P* values less than .05 were considered to be statistically significant.

## Results

### Generation of RGS18 mutant mice

To determine the role of RGS18 *in vivo*, RGS18 mutant mice were generated. In order to disrupt the RGS18 gene by homologous recombination in ES cells, a targeting vector was designed, with a selectable neomycin resistance cassette (Figure S1). After electroporation of the targeting vector into 129S2/SvPas ES cells, several independent clones exhibiting the expected homologous disruption of *RGS18* were used to generate chimeric mice by blastocyst injection (see Materials and Methods). Chimeric males were used for germline transmission of the targeted RGS18 allele. Adult mice carrying disrupted alleles of the RGS18 gene were viable, healthy, and displayed no visible physical abnormalities. The production colony provided normal ratios of WT and knockout males and females. The MCI performed the phenotype analysis of RGS18-/- mice; the metabolic or functional assessments performed are described in the “Information S1” document: metabolic exploration (Table S1), cardiovascular investigations (Table S2), necropsy and histological analysis (Table S3), and behavioral characterization (Table S4). No significant difference was observed between WT and RGS18-KO mice (data not shown), except during behavioral phenotyping where mutant mice displayed behavior that could be interpreted as an increase in reactivity/anxiety and increased pain sensibility (see Table S5, Figure S2 and Figure S3).

### *In silico* analysis of RGS18 promoter

To further characterize RGS18 function, an *in silico* prediction of regulatory elements in the proximal promoter region was performed. 18 transcription factor binding sites conserved in at least 4 species were identified, and 11 of them are described as regulators of hematopoietic gene expression. More specifically, within the 200 bps from the transcriptional start site, 5 cis-regulatory elements corresponding to transcription factors, known to play a role in the development of hematopoietic cell lineages, were found (Figure S4). These binding sites correspond to the transcription factor GATA1, described as essential for erythroid and megakaryocyte development; RUNX1, (located close to GATA1) whose collaboration with GATA1 is required for megakaryocyte differentiation [21]; EVI1, frequently found in myeloid leukemias and whose expression is also associated with progression of megakaryocyte differentiation [22]; STAT3, which among its numerous diverse gene functions has been described to play a role in early stage of megakaryopoiesis too [23]; and MEIS1, known to play an important role in megakaryocytic-specific genes [24]. The identification of these predicted regulatory elements, associated with their co-localisation in the proximal promoter of RGS18 suggests a possible regulation of its gene expression during megakaryopoiesis.

### Mice lacking RGS18 present a mild thrombocytopenia

Analysis of peripheral blood samples of RGS18-/- mice and normal littermates for their hematologic profile showed that RGS18-/- mice were moderately thrombocytopenic: a mean of ∼15% reduction in platelet counts was observed in RGS18-/- mice with respect to WT mice (Table 1). All other blood parameters analyzed, including red blood cells and total white blood cells, were normal in RGS18-/- mice. Interestingly, the mean platelet volume was significantly increased from an average of 4.94 µm^3^ in WT mice to 5.05 µm^3^ in RGS18-/- mice. These data suggested that RGS18 might be implicated in megakaryopoiesis/thrombopoiesis.

**Table 1:** Peripheral blood cell counts in WT and RGS18-/- mice.

	WT	RGS18-/-	P values
**Platelets**	1291.3	1119.1	<0.0001 (***)
** MPV**	4.94	5.05	0.0031 (**)
			
**Red Blood cells**	9.53	9.71	NS
			
**White Blood cells**	8.65	9.07	NS
** Lymphocytes**	6.054	6.496	NS
** Monocytes**	0.35	0.37	NS
** Granulocytes**	2.24	2.2	NS

Blood collected by retro-orbital venous puncture was analyzed in a VetABC animal cell counter. All counts are thousands per microliter, except for red blood cells which are millions per microliter. *P<.05; **P<.01; ***P<.001.

**Figure 1:**
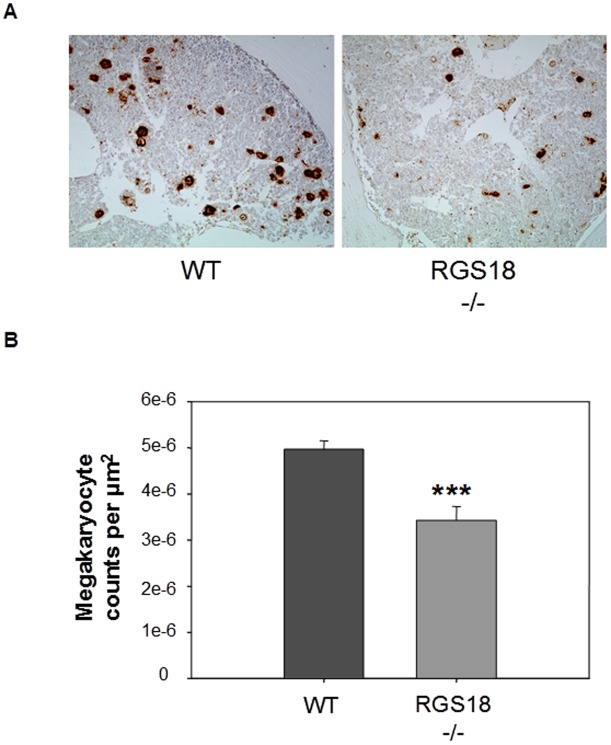
Bone marrow MKs in RGS18-/- mice. (A) Representative vWF immunostaining images of frozen femur sections from 9-week-old WT and RGS18-/- mice. Positively stained MKs from 6 bone marrow sections per mice (n = 3 per group) were scored under a microscope. Images were obtained using ECLIPSE E800 microscope (Nikon, Tokyo, Japan) equipped with a 4x objective (Nikon Eclipse E800). The number of positive vWF MKs was counted by using the 20x objective. Morpho Expert software (Explora Nova, La Rochelle, France) was used to acquire and process images. (B) Cumulative analysis of the numbers of MKs per square millimeter is shown. ***P<.001.

One of the direct causes of thrombocytopenia is a reduction in the number of megakaryocytes (MK) in the bone marrow. We therefore examined MK counts in femora after staining of bone marrow sections for Von Willebrand Factor, a sensitive marker for identification of marrow MKs [25]. As shown in Figure 1, the number of MKs per µm^2^ was markedly decreased in RGS18-/- mice.

In mice, in addition to bone marrow, the spleen is an important alternative hematopoietic organ. Indeed, MKs rarely appear in the spleen of normal adult mice, but are often found in the spleen when megakaryopoiesis in bone marrow is disrupted [26], [27]. Therefore, we also examined the number of splenic MKs in RGS18-/- mice. Although MK counts were slightly increased in the spleens of RGS18-/- mice (0.87±0.07, n = 6, in RGS18-/- mice versus 0.72±0.08, n = 6, in WT mice), the difference was not found to be statistically significant (Figure S5).

### Defective megakaryopoiesis in RGS18-/- mice

**Figure 2:**
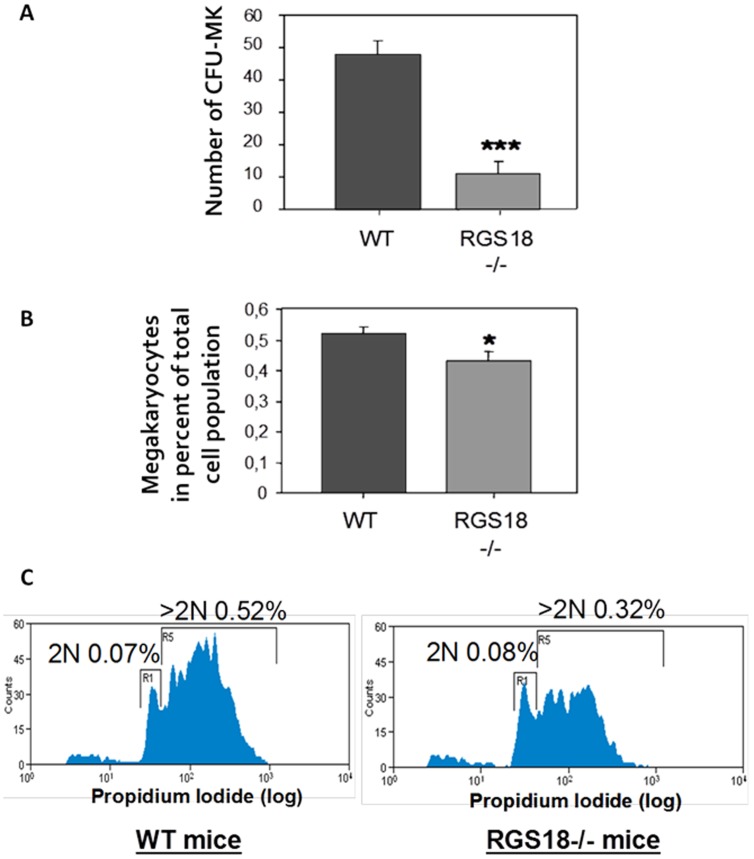
RGS18-/- mice present a defective megakaryopoiesis. (A) *In vitro* MK maturation. Lin^-^ bone marrow cells (5×10^4^ cells/chamber) from 9/10-week-old WT and RGS18-/- mice (n = 5 per group) were cultured in Mega-Cult-C media containing 50 ng/mL rhTPO, 10 ng/ml rmIL3, and 3 ng/ml rmIL6 in double-chamber slides at 37°C for 11 days. Slides were then dehydrated and stained with CD41. Slides were scored microscopically, and MK colonies (CFU-MKs) were defined as colonies containing at least 3-4MKs. (B) *In vivo* MK maturation. Bone marrow cells from 9-week-old WT and RGS18-/- mice (n = 6 per group) were stained for CD42b, and MKs were identified by flow cytometry on the basis of size and CD42b positivity. Percentages of total bone marrow cell population are shown. (C) MK ploidy analysis. Bone marrow cells from 9-week-old WT and RGS18-/- mice (n = 6 per group) were stained for CD41 in presence of propidium iodide (PI). MKs were identified by flow cytometry on the basis of size and CD41 positivity. DNA cell content was evaluated on the basis of PI fluorescence intensity. Percentages of total bone marrow cell population with DNA content >2N are shown. *P<.05; **P<.01; ***P<.001.

To further characterize megakaryopoiesis in the absence of RGS18, MK progenitor cells from bone marrow of RGS18-/- and WT mice were first assayed in clonogenic culture. *In vitro* colony assays in presence of a cocktail of cytokines regulating megakaryopoiesis showed that the number of MK progenitors (CFU-Meg) was clearly higher in WT mice than in RGS18-/- mice (Figure 2A), indicating that RGS18 deficiency in MK progenitors affects their *in vitro* maturation potential.

To confirm these results, we next examined the *in vivo* maturation potential of bone marrow MKs. Bone marrow cells were stained for CD42b and MKs were identified by flow cytometry. We observed that the level of MKs from bone marrow of WT mice was in accordance with litterature data (<1%). However, the percentage of MKs was significantly decreased in RGS18-/- mice (Figure 2B), as previously observed in the histologic analysis of bone marrow sections.

Finally, we analyzed the ploidy distribution of bone marrow MKs. Ploidy, a distinctive characteristic of differentiated MKs, was measured by flow cytometry using propidium iodide fluorescence. Interestingly, we found that the frequency of early MK progenitors with 2N DNA content in RGS18-/- mice was similar to that of WT mice, while the proportion of more committed MK precursors with >2N DNA content was markedly decreased in RGS18-/- mice (Figure 2C), indicating a direct correlation between RGS18 and MK maturation.

### RGS18 deficiency induces a defect in platelet generation after acute thrombocytopenia

**Figure 3:**
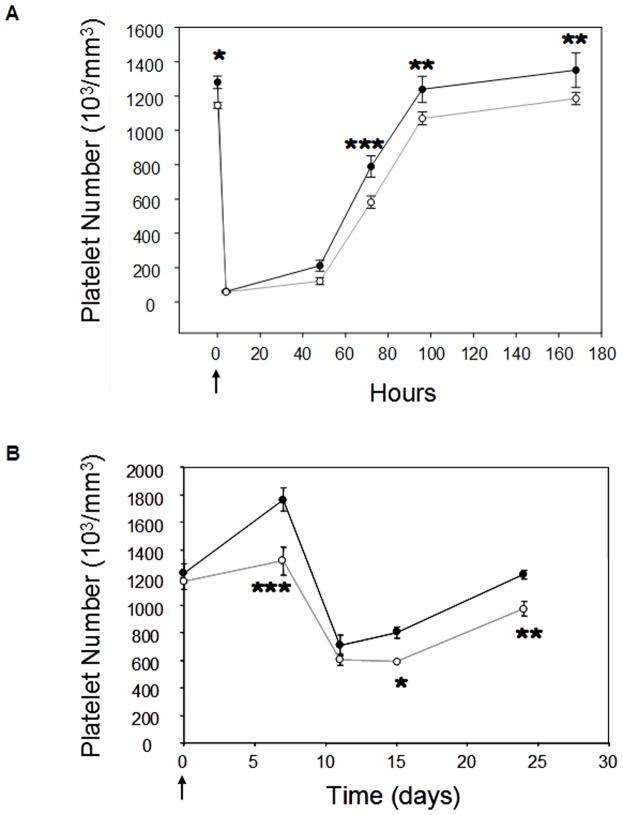
RGS18-/- mice present a defective thrombopoiesis after acute thrombocytopenia. (A) Complement-mediated immune thrombocytopenia. 10-week-old WT (n = 8; black circle) and RGS18-/- mice (n = 8; open circle) were given a sterile intraperitoneal injection of anti-mouse αGpIb antibody (2 µg/g of mouse) at T0 (Arrow). Platelet counts were measured at 4, 48, 72, 96, and 168 hours after injection. (B) Busulfan-induced thrombocytopenia. Busulfan was injected to 9/13-week-old WT (n = 15; black circle) and RGS18-/- mice (n = 14; open circle) intraperitoneally at 30 mg/kg (day 0; arrow). Platelet counts were measured at 7, 11, 15, and 24 days after injection. *P<.05; **P<.01; ***P<.001.

To evaluate the involvement of RGS18 in thrombocytopoietic activity *in vivo*, experiments of acute thrombocytopenia were conducted in RGS18-/- mice and their normal littermates. In the first model, a complement-mediated immune thrombocytopenia was induced by an intraperitoneal injection of an anti-mouse GpIb antibody, based on the method of Nieswandt et al. [28]. As shown in Figure 3A, GpIb antibody injection led to a dramatic decrease (>95%) in platelet counts within 4 hours in RGS18-/- and WT mice. Platelet recovery was measured at 48 h, 72 h, 96 h, and 168 h post-injection. As previously observed, recovery of platelet counts was achieved between 96 h and 168 h, and a defective platelet recovery was clearly observed in RGS18-/- mice (Figure 3A).

In the second model of thrombocytopenia, mice were myelosuppressed by intraperitoneal injection of busulfan, a cancer chemotherapy agent. The busulfan-treated mice showed platelet nadir around day 11, and then the platelet number gradually recovered to approximately 100% by day 24 in WT mice (Figure 3B). RGS18-/- mice showed a lower platelet number at the nadir and a significantly reduced platelet recovery over the experimental period compared to WT mice (Figure 3B).

### RGS18-/- mice display a platelet destructive disorder

**Figure 4:**
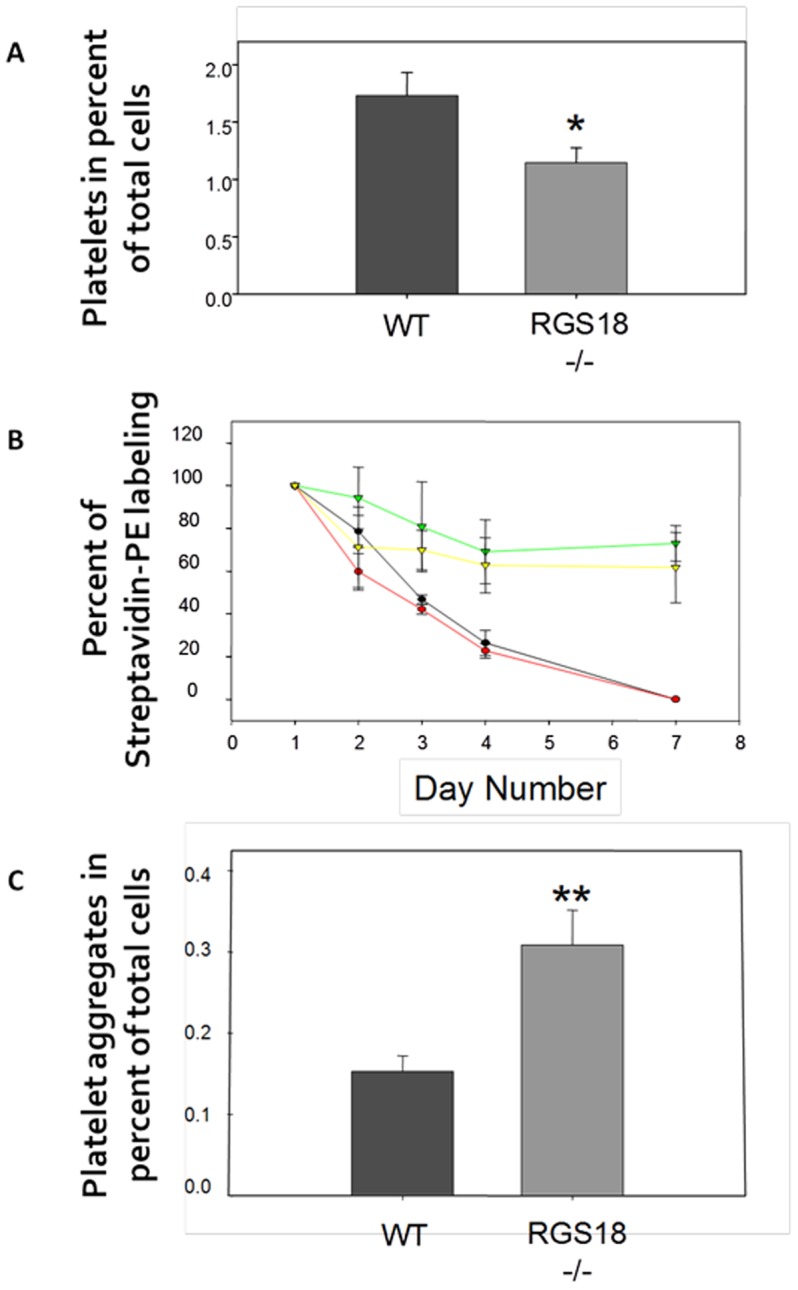
Rate analysis of platelet removal from blood. (A) Splenic sequestration of platelets. Crushed spleens from 9/13-week-old WT and RGS18-/- mice (n = 6 per group) were stained for CD42b, and platelets were identified by flow cytometry on the basis of size and CD42b positivity. Percentages of total splenic cell population are shown. (B) Clearance of platelets. Blood cells from 7-week-old mice were biotinylated *in vivo* by infusion of NHS-biotin, and then blood samples were collected at the time indicated. Biotinylated platelets from WT (black line) and RGS18-/- (red line) mice (n = 9 per group) were identified by flow cytometry on whole blood using PE-Streptavidin and CD41 staining. The stability of the biotinylation was assessed by examining the *in vivo* biotinylated CD41^-^ blood cells in WT (green line) and RGS18-/- (yellow line) mice (n = 9 per group). (C) Spontaneous platelet aggregation. Whole blood cells from 9/11-week-old WT and RGS18-/- mice (n = 12 per group) were stained for CD42b, and platelets were identified by flow cytometry on the basis of size and CD42b positivity. Platelet aggregates were monitored by extending the platelet gate so as to include the platelet “smearings” having elevated FSC and FITC fluorescence. Percentages of total cell population are shown. *P<.05; **P<.01.

In addition to a defective platelet production by the bone marrow, thrombocytopenia could also reflect an increased rate of platelet removal from the blood. Abnormalities in different processes can lead to this, i.e. increased trapping of platelets by the spleen, diminished platelet survival, or platelet consumption. Thus, we first examined if the observed thrombocytopenia in RGS18-/- mice could be caused by a splenic sequestration of the platelets. As shown in Figure 4A, the spleens of the RGS18-/- mice did not contain an increased number of platelets. Platelet counts were significantly decreased compared with those of the WT mice (p = 0.031), in a parallel with the respective number of circulating platelets in WT and RGS18-/- mice. We consistently did not observe any splenomegaly in the RGS18-/- mice (data not shown).

To determine whether alterations in platelet half-life could contribute to thrombocytopenia in mice lacking RGS18, *in vivo* labeling studies of blood cells were performed by biotinylation. Flow cytometry analysis revealed no difference in the rate of platelet clearance from the circulation in RGS18-/- mice compared to WT controls (Figure 4B), indicating that the lower level of circulating RGS18-/- platelets is not the result of diminished platelet survival.

Finally, we investigated whether platelets from RGS18-/- mice could be consumed by spontaneous aggregation. Spontaneous platelet aggregation (SPA) was evaluated by flow cytometry on whole blood with no platelet agonists added. Analysis of circulating platelet aggregates showed that the RGS18-/- mice presented more spontaneous platelet aggregates than WT mice (Figure 4C), suggesting that the observed thrombocytopenia in RGS18-/- mice could be the reflect of both a defective megakaryopoiesis and a spontaneously enhanced platelet aggregability.

### RGS18 deficiency induces a prothrombotic phenotype in mice

**Figure 5:**
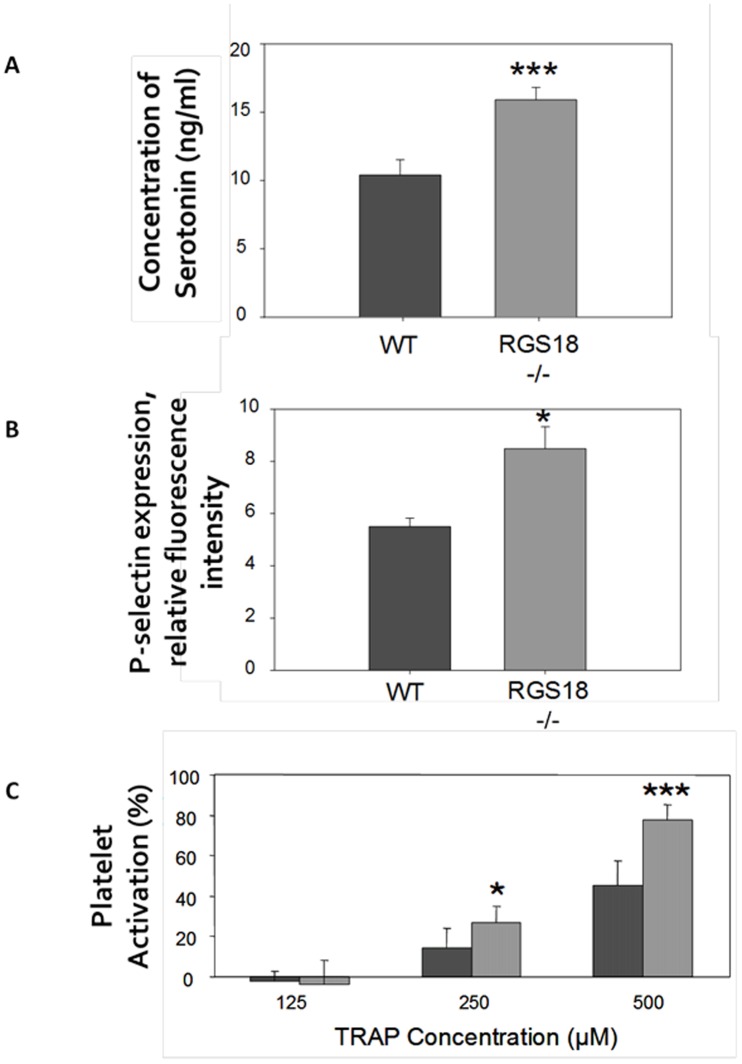
RGS18 deficiency induces a prothrombotic phenotype in mice. (A) Serum levels of serotonin. Blood from 10-week-old WT and RGS18-/- mice (n = 9 per group) was collected, and the quantitative determination of serotonin in serum was performed by enzyme immunoassay (ELISA). Concentrations of serotonin (ng/ml) are shown. (B) P-selectin (CD62P) expression on platelets. Whole blood cells from 12-week-old WT and RGS18-/- mice (n = 6 per group) were stained for CD62P, and platelets were identified by flow cytometry on the basis of size and CD42b positivity. Fluorescence intensities of platelet CD62P are shown. (C) Platelet activation in response to TRAP. Platelet-rich plasma (PRP) from 9/11-week-old WT and RGS18-/- mice (n = 6 per group) was prepared, and incubated at 37°C during 3 minutes with different concentrations of TRAP (µM). Platelet activation was measured by flow cytometry detection of activated integrin αIIbβ3. Percentages of platelet activation are shown. *P<.05; ***P<.001.

To further investigate whether RGS18-/- mice could present thrombogenic disorders, we first analyzed the basal platelet activation state by measuring platelet activation markers. Serotonin has been shown to play a central role in the haemostatic process, and its release is considered to be the most reliable biological assay for platelet activation [29]. The quantitative determination of serotonin in plasma by enzyme immunoassay measurement showed increased levels in RGS18-/- mice, with concentrations being approximately 53% higher than those measured from plasma samples of the WT mice (Figure 5A). CD62P (P-selectin) is a constituent of alpha granules and is released to the platelet surface upon activation, a process parallel to secretion of serotonin [30]. Basal platelet activation was thus assessed by flow cytometry on whole blood measuring surface CD62P expression with no platelet agonists added. Figure 5B shows a mean of ∼54% increase in basal CD62P expression on RGS18-/- platelets with respect to WT platelets, indicating that RGS18 deficiency primes platelet activation even in the absence of an added platelet agonist.

By analyzing basal and TRAP-activated platelets using 2-DE gel electrophoresis, RGS18 has recently been found to be phosphorylated on Ser49 in response to TRAP activation [31]. To determine whether RGS18 could also regulate PAR signaling in platelets, we next examined the platelet activation responses to TRAP and thrombin in WT and RGS18-/- mice. Platelet activation was assessed by flow cytometry measuring surface expression of activated integrin αIIbβ3. As shown in Figure 5C, murine TRAP (PAR-4 peptide activator) dose-dependently induced an activation-dependent conformational change in integrin αIIbβ3 on platelets from WT littermates, and an enhanced response was observed in RGS18-/- mice as early as the concentration of 0.25 mM of mTRAP. Similarly, the response to thrombin was found to be increased in RGS18-/- mice, as assessed using *in vitro* platelet aggregation assays (data not shown).

### RGS18-/- mice show enhanced platelet function *in vivo*

**Figure 6:**
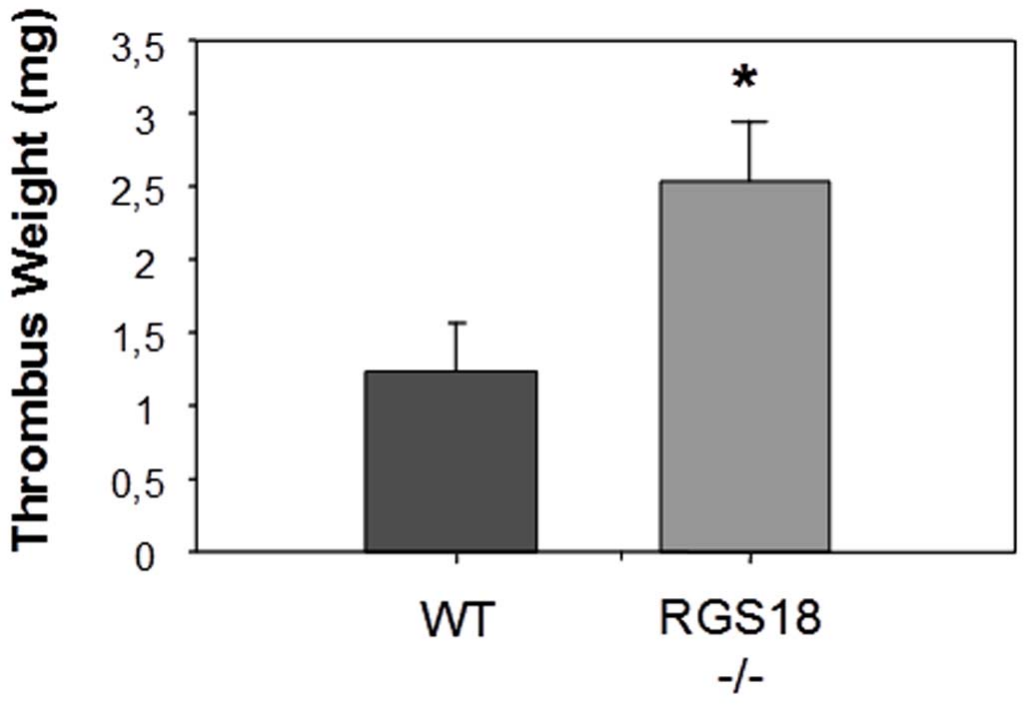
RGS18 deficiency increases thrombus formation at sites of arterio-venous shunt *in vivo*. An arterio-venous shunt was achieved as described on 11/13-week-old WT and RGS18-/- mice (n = 14 per group) and thrombus weight was determined after 10 minutes of blood circulation. *P<.05.

Platelets are known to play a central role in both arterial and venous thrombosis. To determine whether RGS18 deficiency affects platelet function *in vivo*, we used the silk thread arterio-venous shunt thrombosis model, which has been characterized as a “mixed” thrombosis model in rats [32]. In this model, both the activation of platelets (often linked to arterial thrombosis) and the coagulation cascade (commonly associated with venous thrombosis) are known to contribute to the thrombus formation. Since RGS18-/- platelets displayed an enhanced basal aggregability, thrombus formation was measured after a very short time of blood circulation. Analysis of thrombus weight after a 5 min-blood flow showed that RGS18 deficiency markedly increased platelet accumulation, with thrombi being more than 2 times larger in RGS18-/- mice than those of WT mice (Figure 6).

In agreement with the prothrombotic phenotype of the RGS18-/- mice, there was no evidence of spontaneous bleeding in these mice (data not shown).

## Discussion

RGS18 has been described in 2001 as a myeloerythroid lineage-specific regulator of G-protein signaling, highly expressed in MKs and platelets. Several studies report that the RGS18 transcript is specifically detected in haematopoietic progenitor and myeloerythroid lineage cells, and that they are most highly abundant in MKs and platelets [10]–[12]. In accordance with this expression pattern, our results show that RGS18 is important for MK biology and that RGS18 deficiency produces a defective megakaryopoiesis, affecting the maturation potential of MK progenitors *in vitro* as well as *in vivo*. Moreover, RGS18 deficiency induces a chronic thrombocytopenia in mice and a defect in platelet recovery and formation after acute thrombocytopenia. Together, these results suggest that RGS18 plays a physiological role in hematopoiesis, in particular taking an active part in megakaryopoiesis and platelet generation. It is interesting to note that Louwette and colleagues recently reported that lentiviral RGS18 overexpression during *in vitro* differentiation of mouse Sca1^+^ hematopoietic stem cells induced an increase in MK proliferation and that RGS18 depletion in zebrafish resulted in thrombocytopenia [33].

In 2005, Berthebaud and colleagues proposed that RGS16 might be a negative regulator of SDF-1/CXCR4 signaling in MKs [13]. They observed that overexpression of RGS16, but not of RGS18, in MO7e cells inhibited SDF1-induced migration, and down-regulation of RGS16 by RNA interference in MO7e cells and in primary MKs promoted SDF1-induced chemotaxis. In contrast with their results obtained with RGS18, we did indeed observe a functional role of RGS18 in MK migration (Figure S6). Actually, RGS18 downregulation by RNA interference in Dami cells comes with an increase of SDF1-induced Dami cell migration. The apparent discrepancy between these two sets of results could arise not only from the use of different megakaryoblastic cell lines, but also from the different technical strategies used to evaluate the role of RGS18 in MK cell migration – namely RGS18 overexpression versus silencing. To date, RGS18 expression levels in MK cells have been always reported to be strong and much higher than RGS16 expression levels [12], [13], [15]. Therefore, overexpressing RGS18 in cells where its expression level is already very strong might not be the most appropriate approach for observing subsequent effects. Furthermore, our results are well supported by a study from Nagata and colleagues who found that SDF-1 binding to its receptor CXCR4 affected the binding capacity of RGS18 to Gαi, but not Gαq, in MKs [10]. Based on these different observations, RGS18 might control MK migration to regulate megakaryopoiesis by attenuating CXCR4 signaling, suggesting that a functional redundancy among RGS16 and RGS18 might exist in MKs.

RGS1, 2, 3, 6, 9, 10, 16, 18, and 19 transcripts are shown to be detected in human platelets [14] and RGS2, 3, 5, 6, 10, 14, 16, and 18 transcripts are present in rat platelets [15]. At the protein level, only RGS10 and RGS18 have been detected in human platelets [31], [34]. Functionally, these two RGS proteins have been found: (a) to be differentially phosphorylated after TRAP stimulation of platelets [31], (b) to be associated with spinophilin (SPL) in resting platelets [34], and (c) to be released from SPL after platelet activation by thrombin [34]. Therefore, a functional redundancy among RGS family members might exist in platelets. At the bone marrow level, mRNA transcripts of RGS1, 2, 8, 10, 12, 14, 16, 18, and 19 are detected in mice, and no difference in their expression level is observed in extracts from either RGS18-deficient mice and WT mice (Figure S7). Because RGS18-deficient mice are viable, elucidating whether RGS10 partly compensates for RGS18 function in our RGS18 knockout model is a question that will need to be addressed.

In addition to the regulatory role of RGS18 in MK biology, our results show that RGS18 deficiency produces a gain of function phenotype in platelets, shifting the dose-response curve for platelet aggregation induced by thrombin to the left *in vitro* and increasing thrombus formation at sites of arteriovenous shunt *in vivo*. Moreover, we also show that RGS18 deficiency is sufficient to prime platelet activation even before an agonist is added. Collectively, these observations show for the first time the functional significance of RGS18 in platelets and suggest that its physiological role is to limit platelet activation and accumulation during thrombus formation.

In accordance with our results, Signarvic and colleagues recently demonstrated the active role of RGS proteins in regulating platelet responsiveness [17]. Platelets from mice with a G184S substitution in Giα2 that blocks RGS/Gi2 interactions, showed increased responsiveness to agonists *in vitro* and increased accumulation after vascular injury *in vivo*. This increased responsiveness was not limited to Gi2-coupled receptor agonists such as ADP. Giα2 (G184S) substitution also produced increased responses to PAR4 and TxA2 receptor agonists, although signaling downstream of these receptors in platelets is usually attributed to Gq and (to a lesser extent) G12 family members rather than to direct activation of Gi family members. In contrast to these results, we observe that RGS18 deficiency produces a pathway-selective gain of function in platelets. RGS18-deficient platelets respond significantly better than controls at low concentrations of either thrombin (PAR-1 receptor agonist) or TRAP (PAR-4 receptor agonist), whose receptors are both Gq-coupled receptors. On the other hand, RGS18 deficiency does not produce a shift in the dose-response curve for platelet aggregation induced by ADP (Figure S8). The Gi-coupled P2Y12 receptor is the major ADP receptor in platelets, and an increased response of RGS18-deficient platelets at suboptimal ADP concentration is what would be expected if RGS18 protein restrains this Gi-dependent signaling pathway. In investigating possible changes induced by RGS18 deficiency in platelet responsiveness not related to agonists coupled to G-protein pathways, we also found no difference in platelet aggregation induced by collagen between RGS18-deficient and WT platelets (Figure S8). Thus, we can assume that RGS18 retains specificity for pathways known to be mediated by Gq and does not spill over to predominantly Gi-driven events in platelets. To further establish that RGS18 regulation of platelet function is strictly confined to Gq-mediated events, it will be important to evaluate its role in RGS18-deficient platelet responsiveness to TxA2.

Nevertheless, our assumption is strongly supported by latest results of Ma and colleagues who described an association of spinophilin (SPL) with RGS18 in resting platelets and more especially an agonist-selective dissociation of this complex during platelet activation [34]. In their study, dissociation of the SPL/RGS18 complex occurred when human platelets were incubated with the two potent activators of Gq-mediated events, thrombin and TxA2, but not in presence of ADP or collagen. Similarly, we observed an increase of platelet responsiveness when RGS18-deficient platelets are incubated with thrombin or TRAP, but not in presence of ADP or collagen. Both studies converge on the idea that RGS18 might at least control PARs signaling. In 2004, Garcia and colleagues had already observed that PAR receptor signaling induced the phosphorylation of Serine 49 of RGS18 in platelets [31]. More recently, Gegenbauer and colleagues also showed that RGS18 is phosphorylated on S49 and S218 in platelets, and that phosphorylation of S49 increased with platelet activation by thrombin [35], [36]. Furthermore, they also showed that RGS18 inhibited Gq-mediated Ca^2+^ release from intracellular stores. In agreement with this, we observe, in the megakaryocytic Dami cells, that downregulation of RGS18 by RNA interference strongly increases intracellular Ca^2+^ release as early as the concentration of 0.1 UI/ml of thrombin (Figure S9). Altogether these data suggest that RGS18 might control calcium signaling to limit platelet activation by attenuating Gq signaling pathways in platelets, in particular that of PAR receptors.

In conclusion, by generating RGS18 knockout mice for the first time and describing their phenotype, we have helped to advance in the understanding of the role of RGS proteins on MK differentiation and platelet generation *in vivo*. RGS proteins play essential regulatory roles in the signaling of G protein-coupled receptors and display remarkable specificity and selectivity in their regulation of GPCR-mediated physiological events. GPCRs constitute the largest family of receptors in the genome and are the targets for at least 50% of current medicines. For many G protein-coupled receptors, intense efforts to develop orthosteric drugs have failed to yield highly selective ligands. In recent years, major advances have been made and established allosteric GPCR modulators as a novel approach to regulate this important class of drug targets. In particular, these allosteric modulators have provided novel tools and drug leads for multiple receptors for which efforts aimed at discovery of orthosteric ligands had been unsuccessful. In parallel, research data have highlighted RGS proteins as attractive targets for the development of potential future therapeutics that would control GPCR signaling in a tissue- or pathway-specific manner. Here we have demonstrated the functional importance of endogenous RGS18 *in vivo*, showing that RGS18 is a key regulator of GPCR signaling in MKs and platelets, controlling both platelet generation and platelet function. Numerous RGS knockout mice have been generated [37]. These models have provided essential information for our understanding of the physiological role of RGS proteins but in many cases, reported effects are modest [38], [39]. In the present study, we describe readily detectable effects in RGS18-deficient mice but nevertheless with only a mild phenotype. The most likely explication is a functional redundancy with other RGS proteins. It is evident that at least RGS10 and RGS16 present some degree of functional redundancy with RGS18 *in vitro* in platelets and megakaryocytes, respectively [31], [34], [13]; which might partially compensate for RGS18 deficiency in the different cellular contexts and thus explaining the minimal phenotype observed in mutant mice. From a clinical perspective, it is not clear whether increasing RGS18 function could protect from cardiovascular or hematological diseases. Yet what can be said at this point with some degree of assurance is that enhancers rather than inhibitors may be more useful in RGS18 drug development; emerging data show reductions in RGS protein expression or function in several pathophysiological states, and strategies to increase RGS function are now emerging [40].

## Supporting Information

Figure S1 **Targeting vector with a selectable neomycin resistance cassette (F_NEO) designed to disrupt the RGS18 gene.**
(TIF)

Figure S2 **Acoustic startle reactivity (A) and pre-pulse inhibition (B) in WT and RGS18-/- mice.**
(TIF)

Figure S3 **Anxiety assessment of RGS18-/- mice using the open field test.**
(TIF)

Figure S4 **In silico analysis of RGS18 Promoter.**
(TIF)

Figure S5 **Splenic MKs in RGS18 -/- mice.**
(TIF)

Figure S6 **RGS18 silencing increases MK cell migration.**
(TIF)

Figure S7 **RGS gene expression in bone marrow extracts.**
(TIF)

Figure S8 **Platelet activation in response to ADP and collagen.**
(TIF)

Figure S9 **Effect of RGS18 silencing on intracellular calcium release in MK cells.**
(TIF)

Table S1 **List of tests performed for metabolic exploration.**
(TIF)

Table S2 **List of tests performed for cardiovascular investigation.**
(TIF)

Table S3 **Necropsy and histological analysis.**
(TIF)

Table S4 **List of tests performed for behavioral characterization.**
(TIF)

Table S5 **Evaluation of pain sensitivity in WT and RGS18-/- mice.**
(TIF)

Information S1 **Supplementary methods and results.**
(DOC)
