# Supplementary figures and images for: Regulator of G-Protein Signaling 18 Controls Both Platelet Generation and Function
Source: PLoS One. 2014 Nov 18;9(11):e113215. doi: 10.1371/journal.pone.0113215 (PMC4236145; doi:10.1371/journal.pone.0113215)

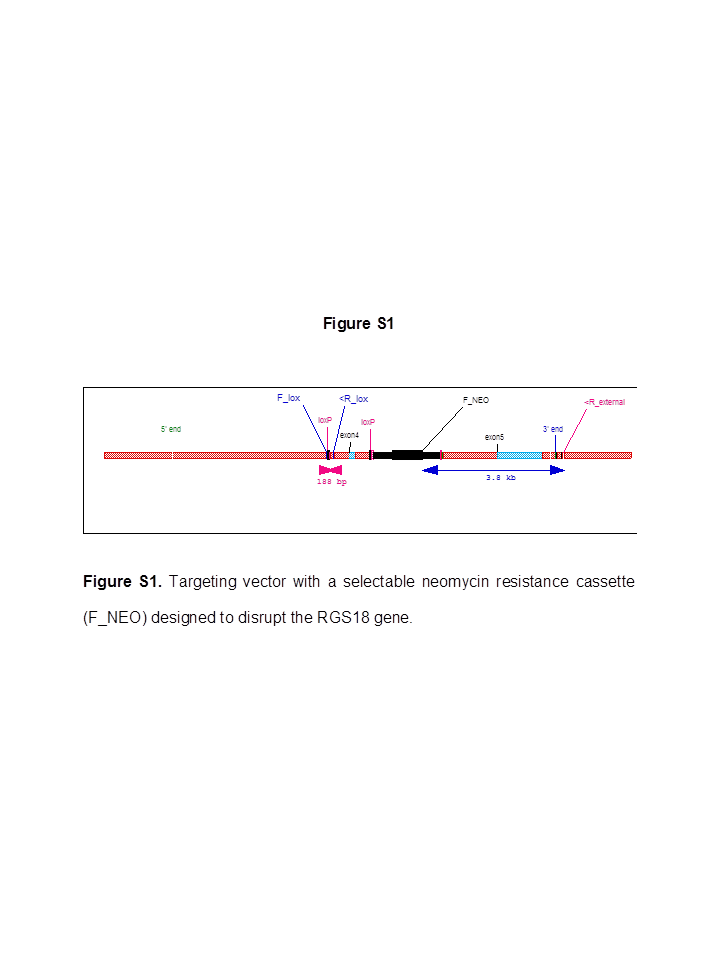

Supplement: Figure S1 — Targeting vector with a selectable neomycin resistance cassette (F_NEO) designed to disrupt the RGS18 gene. (TIF) [file pone.0113215.s001.tif]

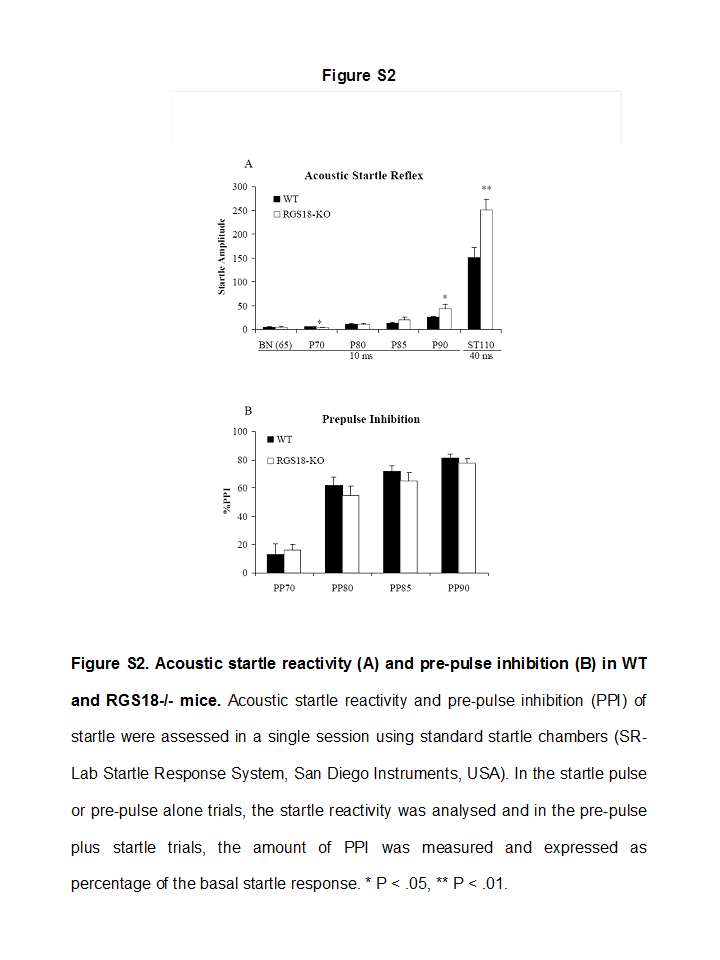

Supplement: Figure S2 — Acoustic startle reactivity (A) and pre-pulse inhibition (B) in WT and RGS18-/- mice. (TIF) [file pone.0113215.s002.tif]

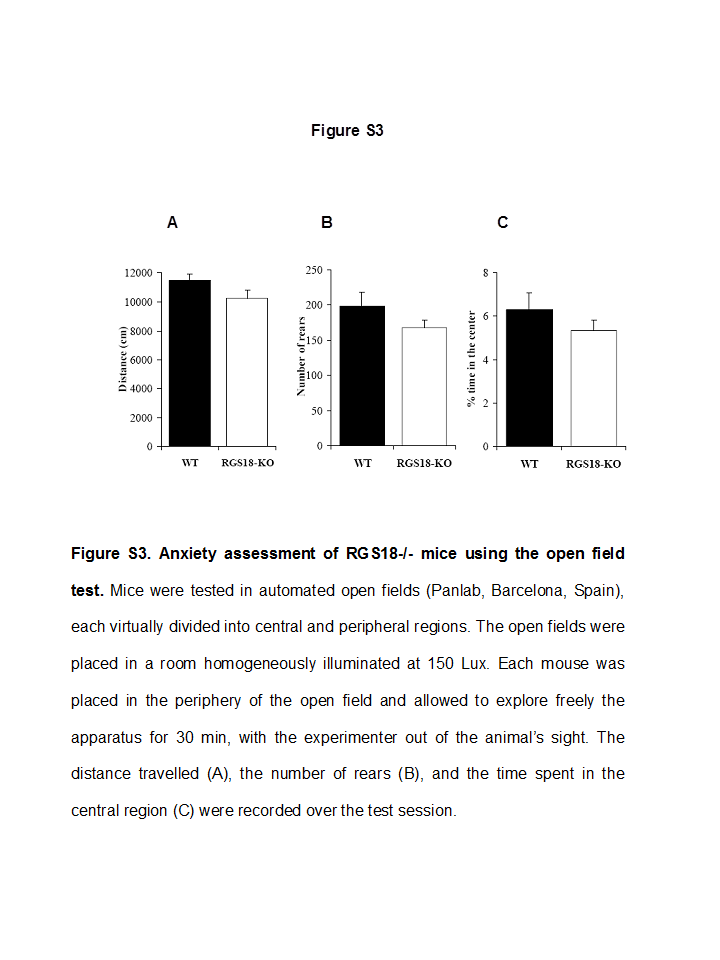

Supplement: Figure S3 — Anxiety assessment of RGS18-/- mice using the open field test. (TIF) [file pone.0113215.s003.tif]

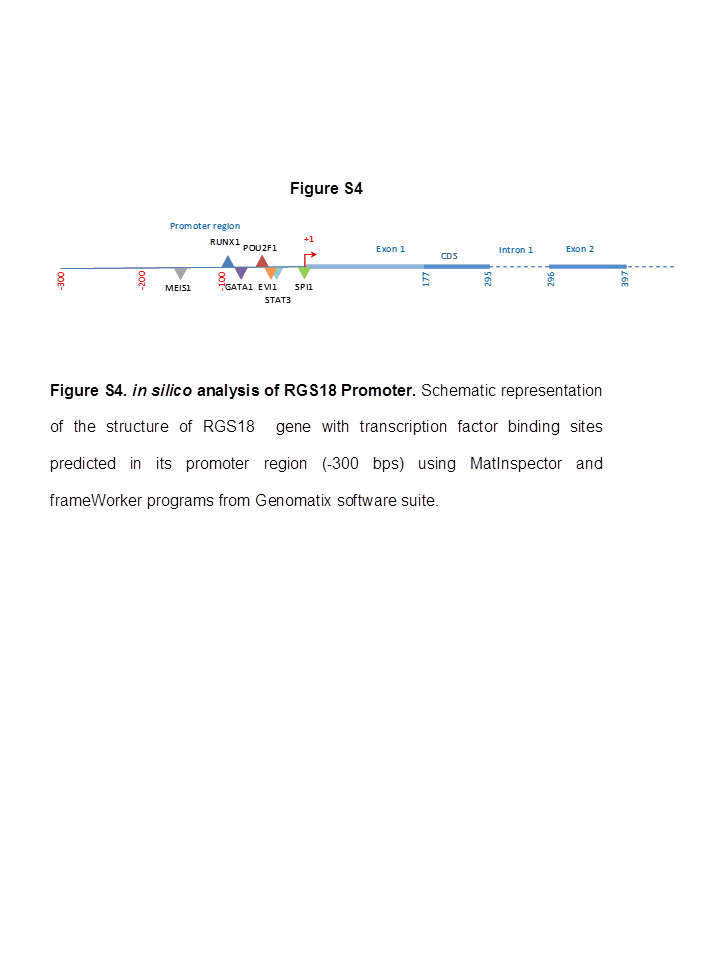

Supplement: Figure S4 — In silico analysis of RGS18 Promoter. (TIF) [file pone.0113215.s004.tif]

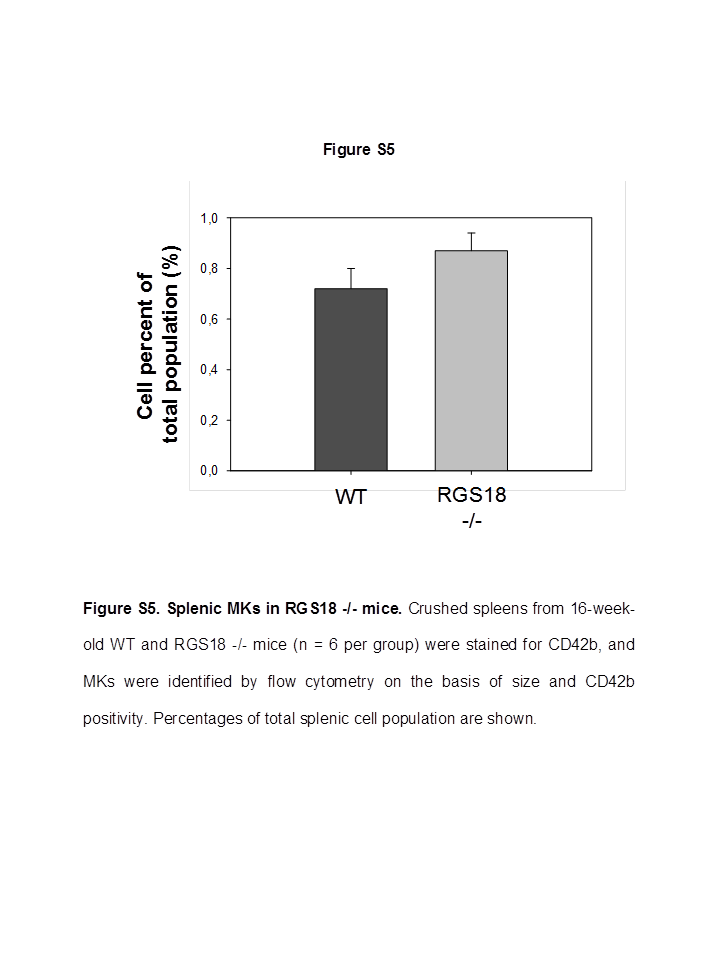

Supplement: Figure S5 — Splenic MKs in RGS18 -/- mice. (TIF) [file pone.0113215.s005.tif]

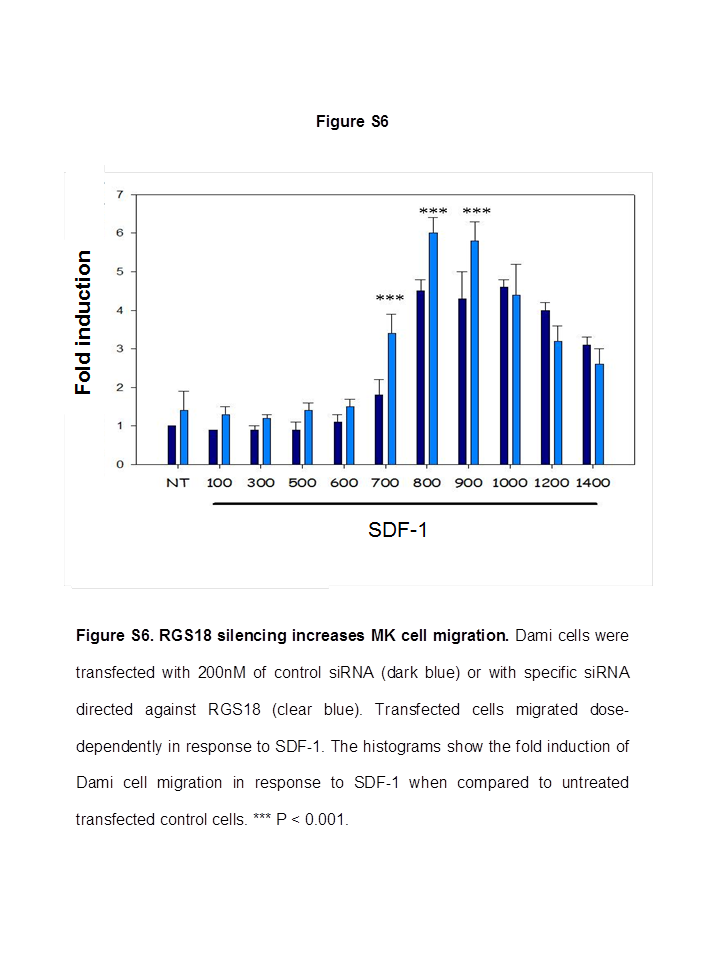

Supplement: Figure S6 — RGS18 silencing increases MK cell migration. (TIF) [file pone.0113215.s006.tif]

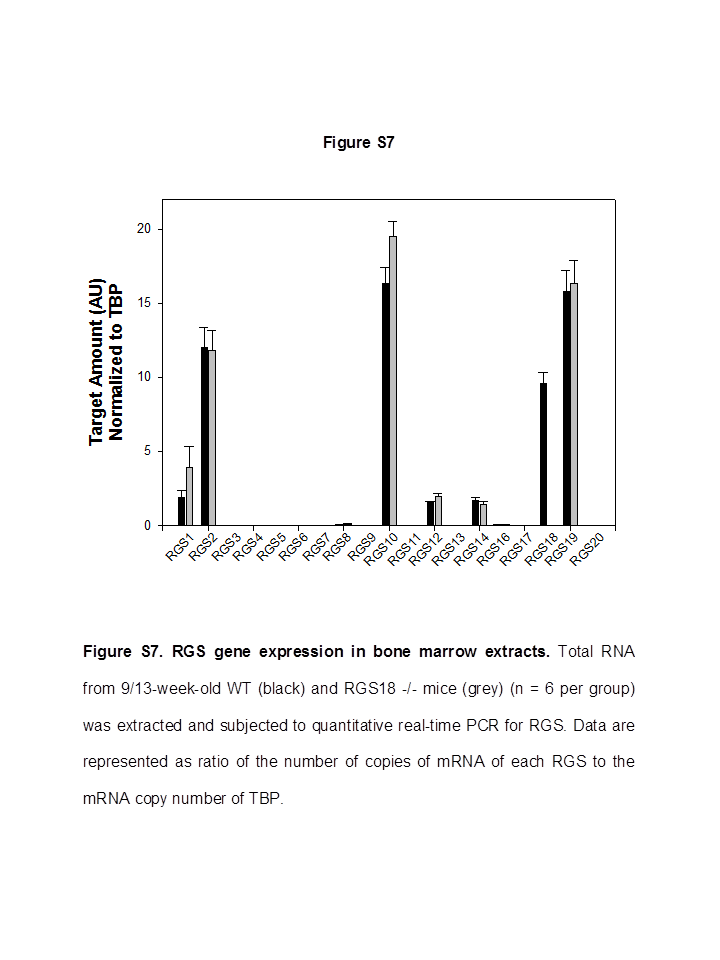

Supplement: Figure S7 — RGS gene expression in bone marrow extracts. (TIF) [file pone.0113215.s007.tif]

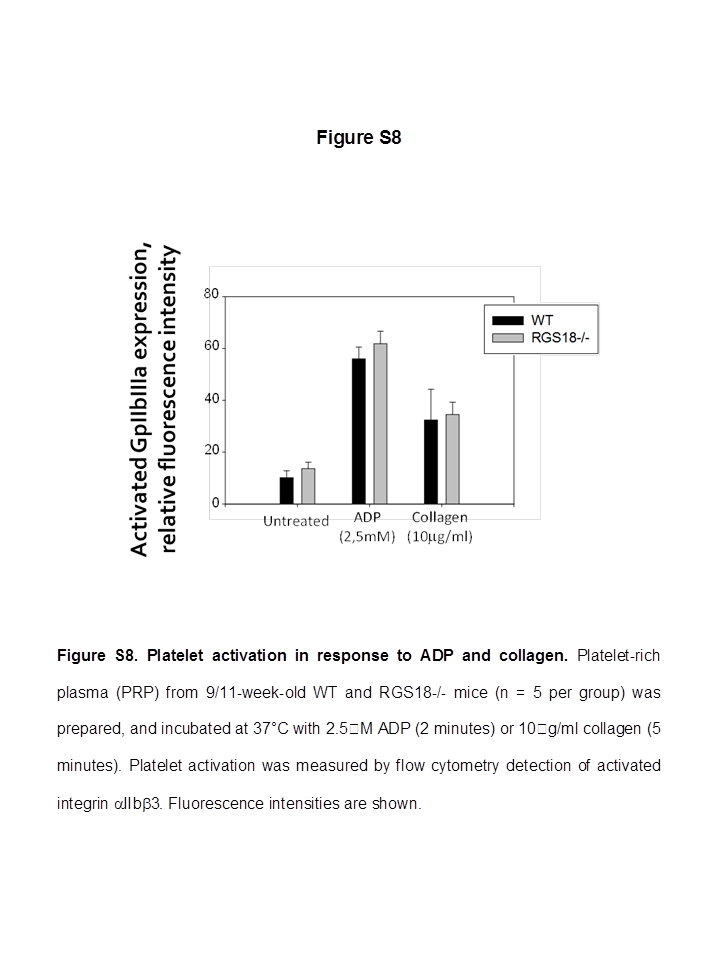

Supplement: Figure S8 — Platelet activation in response to ADP and collagen. (TIF) [file pone.0113215.s008.tif]

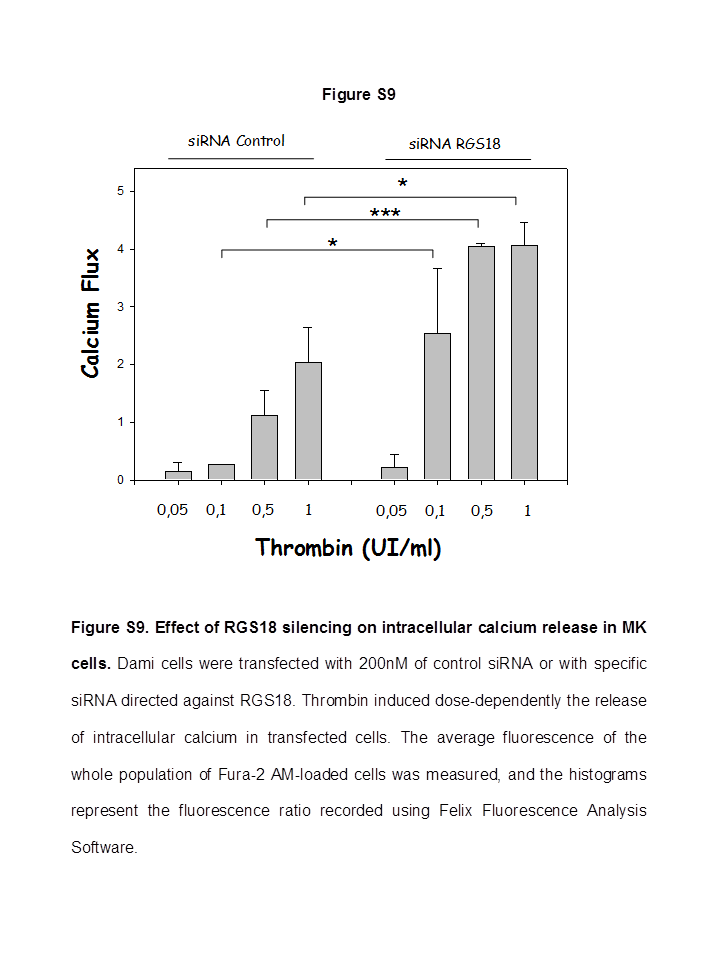

Supplement: Figure S9 — Effect of RGS18 silencing on intracellular calcium release in MK cells. (TIF) [file pone.0113215.s009.tif]

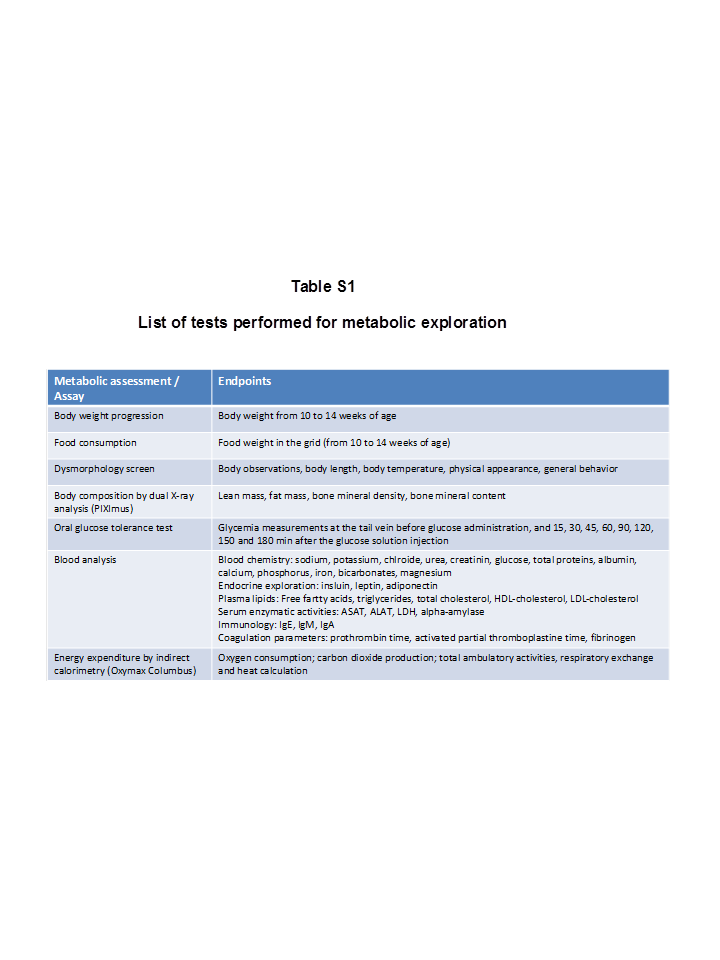

Supplement: Table S1 — List of tests performed for metabolic exploration. (TIF) [file pone.0113215.s010.tif]

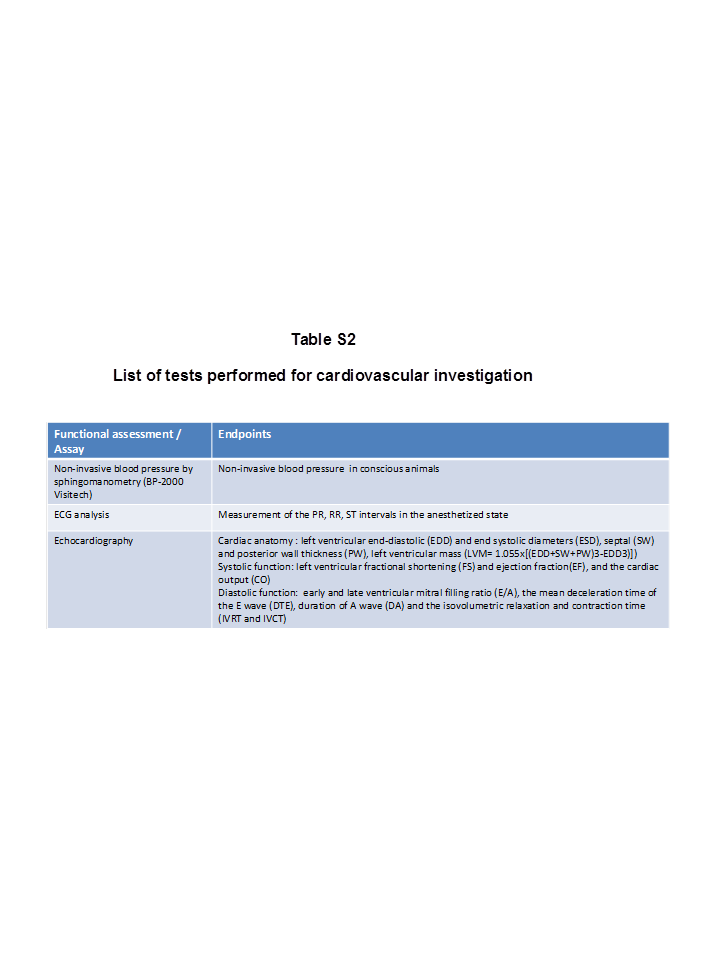

Supplement: Table S2 — List of tests performed for cardiovascular investigation. (TIF) [file pone.0113215.s011.tif]

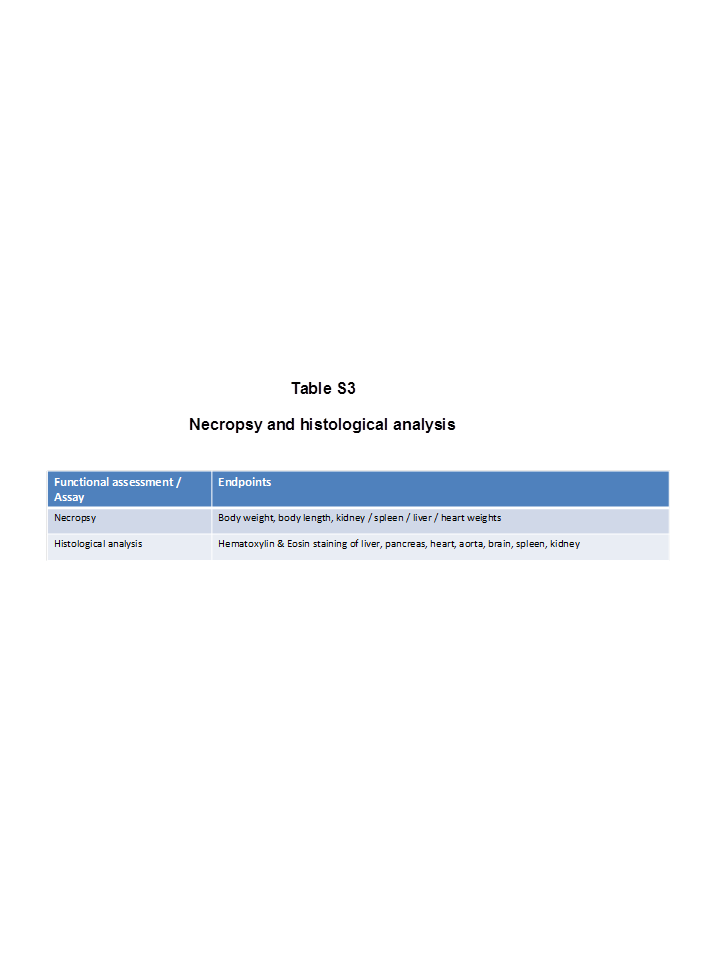

Supplement: Table S3 — Necropsy and histological analysis. (TIF) [file pone.0113215.s012.tif]

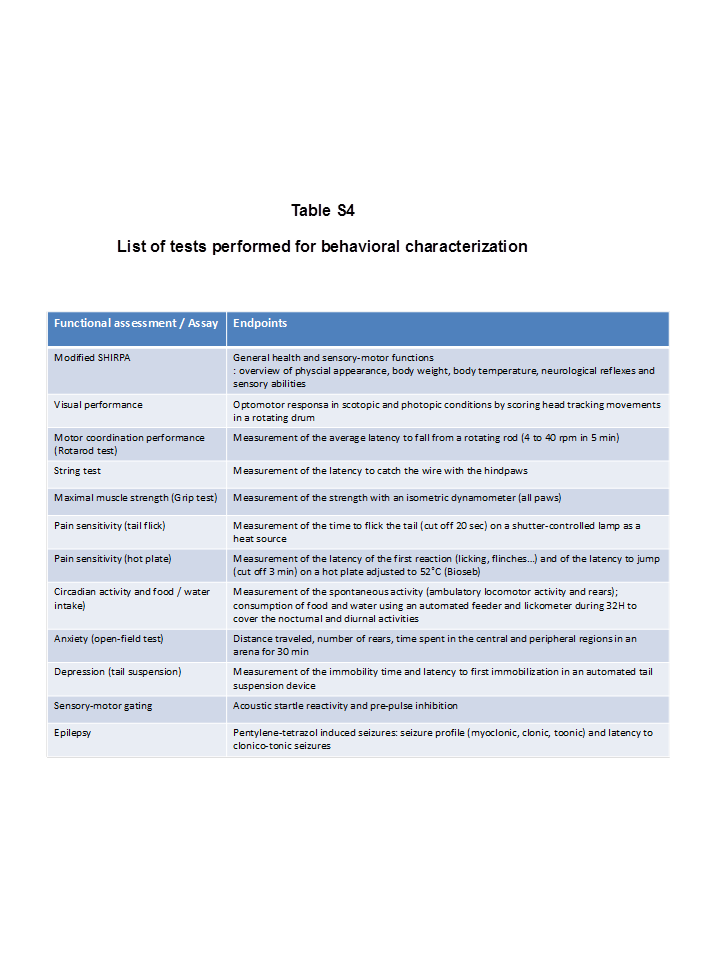

Supplement: Table S4 — List of tests performed for behavioral characterization. (TIF) [file pone.0113215.s013.tif]

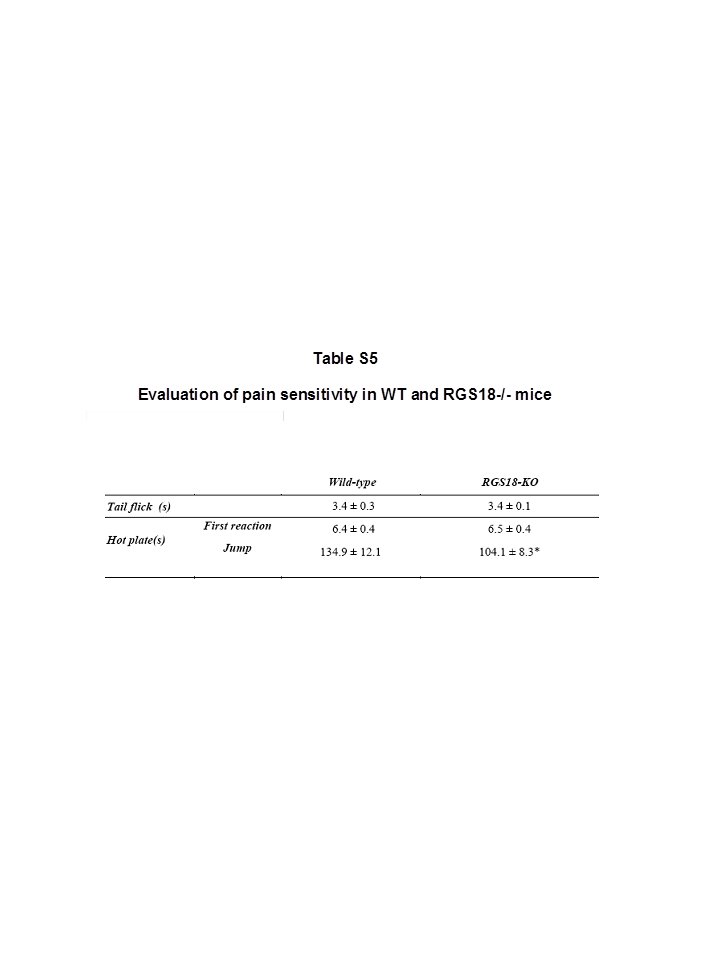

Supplement: Table S5 — Evaluation of pain sensitivity in WT and RGS18-/- mice. (TIF) [file pone.0113215.s014.tif]
