# Supplementary material for: Regulator of G-Protein Signaling 18 Controls Both Platelet Generation and Function
Source: PLoS One. 2014 Nov 18;9(11):e113215. doi: 10.1371/journal.pone.0113215 (PMC4236145; doi:10.1371/journal.pone.0113215)
Supplement: Information S1 — Supplementary methods and results. (DOC) [file pone.0113215.s015.doc]

**Supporting Information 1**

**Supplemental Methods**

**Animals and phenotype analysis at MCI**

All animal procedures described were approved by the CREMEAS, the local ethical committee. All experiments are carried out in accordance with the European Communities Council Directive of 24 November 1986.

Mice were maintained in a room with controlled temperature (21-22°C) under a 12-12 light-dark cycle (light cycle from 7a.m. to 7p.m.) with *ad libitum* access to the water and food (D04 chow diet from UAR, France).

A batch of mice was used to explore the behavioral characteristics of the RGS18-/- mice (only males). Following experiments were performed using mice from 10-week-old to 14-week-old:

*- General health and sensory-motor functions*

The general health and basic sensory motor functions were evaluated using a modified SHIRPA protocol [1]. This analysis provides an overview of physical appearance, body weight, body temperature, neurological reflexes and sensory abilities.

The optomotor response is a robust and reproducible test, allowing evaluation of visual performance in both scotopic and photopic conditions by scoring head tracking movements [2]. Animals were placed in a rotating drum covered with vertical black and white stripes at a spatial frequency of 0.065 cpd. Four measurements of 1 min duration were carried out in scotopic and photopic conditions, during which the drum rotated alternately clockwise and counter-clockwise, with a 30 sec arrest interval. In these conditions, normal animals follow the movement of the drum by moving the head or body whereas animals with altered vision cannot track the stripes.

The rotarod test measures the ability of an animal to maintain balance on a rotating rod (Bioseb, Chaville, France). Mice were given three testing trials during which the rotation speed accelerated from 4 to 40 rpm in 5 min. Trials were separated by 5-10 min interval. The average latency was used as motor coordination performance.

The string test apparatus is a wire stretched horizontally 40 cm above a table. Testing consisted of 3 trials separated by 5-10 min interval. On each trial the forepaws of the animal were placed on the thread. The latency the animal took to catch the wire with its hindpaws was recorded.

The grip test measures the maximal muscle strength (g) using an isometric dynamometer connected to a grid (Bioseb). Each mouse was submitted to 3 consecutive trials immediately after the SHIRPA. Once the animal was holding the grid with its all paws it was slowly moved backwards until it released it. The dynamometer recorded the maximal strength developed.

The tail flick apparatus consists of a shutter-controlled lamp as a heat source (Bioseb). The temperature of the heat bundle was controlled by fixing the Focus and Sensitivity of the system. Three consecutive trials with an interval of about 2-3 min were performed at three different sites of the tail. For each trial, the tail of the animal was placed under the heat source. The time taken by the animal to flick its tail was recorded (cut off 20s).

During the hot plate test, the mice were placed into a glass cylinder on a hot plate adjusted to 52°C (Bioseb). Two trials separated with 5-10 min interval were carried out. For the first trial, the latency of the first reaction (licking, flinches …) was recorded, with a maximum of 30 sec. For the second trial, in addition to the first reaction, the latency to jump was also recorded. A 3 min cut-off was used for the second trial.

*- Circadian activity and food / water intake*

Spontaneous locomotor activity was measured using 24 individual boxes equipped with infra-red captors allowing measurement of ambulatory locomotor activity and rears. The quantity of water and food consumed was measured during the test period using automated pellet feeder and lickometer (Imetronic, Pessac, France). Mice were tested for 32 hours in order to measure habituation to the apparatus as well as nocturnal and diurnal activities.

*- Anxiety and depression*

For the open-field test, mice were tested in automated open fields (Panlab, Barcelona, Spain), each virtually divided into central and peripheral regions. The open fields were placed in a room homogeneously illuminated at 150 Lux. Each mouse was placed in the periphery of the open field and allowed to explore freely the apparatus for 30 min, with the experimenter out of the animal’s sight. The distance travelled, the number of rears, and the time spent in the central and peripheral regions were recorded over the test session. The Number of entries and the percent time spent in center area are used as an indices of emotionality/anxiety.

For the tail suspension test, mice were tested in an automated tail suspension device (MED associates Inc, St Albans, Vermont, USA). Immobility time was monitored through 6-min period in blocks of 2-min intervals, and used as index of despair behavior. Latency to first immobilization was also determined.

*- Sensorymotor gating*

Acoustic startle reactivity and pre-pulse inhibition (PPI) of startle were assessed in a single session using standard startle chambers (SR-Lab Startle Response System, San Diego Instruments, USA). Ten different trial type were used: acoustic startle pulse alone (110-db), eight different prepulse trials in which either 70, 80, 85 or 90-dB stimuli are presented alone or precede the pulse, and finally one trial (NOSTIM) in which only the background noise (65 dB) was presented to measure the baseline movement in the Plexiglas cylinder. In the startle pulse or prepulse alone trials, the startle reactivity was analysed and in the prepulse plus startle trials, the amount of PPI was measured and expressed as percentage of the basal startle response.

*- Epilepsy*

To induce seizures, PTZ was dissolved in saline (0.9 % NaCl) and injected intraperitoneally at the dose of 50 mg/kg. Immediately after injection, the mouse was placed into a new cage and observed for at least 20 min. The seizure profile (myoclonic, clonic, tonic) and the latency to clonico-tonic seizure were recorded.

*- Statistical analysis*

Data were analyzed using unpaired Student t test or repeated measures analysis of variance (ANOVA) with one between factor (genotype) and one within factor (time). Qualitative parameters (e.g. seizure profile, some of clinical observations) were analyzed using Ki2 test. The level of significance was set at p < 0.05.

**Quantitative real-time polymerase chain reaction (PCR) determination of transcript levels**

Total RNA was isolated using the RNeasy Kit (Qiagen GmbH, Hilden, Germany) according to the manufacturer's instructions and contaminating genomic DNA removed by treatment with RNAse-free DNAse kit (Qiagen). Single-stranded complementary DNA (cDNA) was prepared using SuperScript II reverse transcriptase (Life Technologies, Grand Island, NY, USA) according to the manufacturer's instructions. To achieve quantitative gene expression assays for murine RGS1, RGS2, RGS3, RGS4, RGS5, RGS6, RGS7, RGS8, RGS9, RGS10, RGS11, RGS12, RGS13, RGS14, RGS16, RGS17, RGS18, RGS19, or RGS20, PCR reactions were carried out using Assays-on-demand™ Gene Expression Products (PE Applied Biosystems, Weiterstadt, Germany). Quantitative real-time PCR was performed using TaqMan® Universal PCR Master Mix. The reactions were carried out on the ABI PRISM® GeneAmp 7000 Sequence Detection System (PE Applied Biosystems). The copy numbers of the different mRNA transcripts were normalized using TATA box-binding protein (TBP) mRNA levels.

**Cell Culture and Electroporation.** Dami cells were grown in RPMI 1640 medium supplemented with 10% heat-inactivated fetal calf serum, 2mM L-glutamine, 100 units/ ml penicillin and 100 µg/ml streptomycin at 37°C in a humidified atmosphere with 5% CO2. 1.5 × 10^6^ Dami cells were nucleofected® with 200nM of specific siRNA directed against RGS18 (Dharmacon D-008489-01) or control siRNA (Dharmacon D-001230-01) using Amaxa Biosystem’s Cell Line Nucleofector® Kit T program T-20.

**Measurements of cytosolic calcium concentration**. 10^6^ cells were rinsed twice with PSS buffer (5 mM HEPES, 5.6 mM glucose, 145 mM NaCl, 5 mM KCl, 1 mM MgCl2, pH 7.4), then resuspended in 5ml of PBS / 2mM CaCl2 and 0.1% BSA. They were then loaded with 5 ml of 1.5 µM Fura-2 AM dissolved in PBS from a stock solution of 1.5 mM in DMSO containing 20% pluronic acid F-127, at room temperature for 30 min. 40 ml of PBS / 2mM CaCl2 and 0.1% BSA was added and cells were incubated 1 hour at RT to allow complete de-esterification of the dye. Cells were subsequently rinsed twice with PBS / 2mM CaCl2 and 0.1% BSA and the cellular suspension was adjusted to 15 x 10^6^ cells/ml. For measurements of [Ca2+]i, 25 µl of cellular suspension, 30µl of thrombin and 3 ml of PBS / 2mM CaCl2 were added in a cuvette.

The average fluorescence of a whole population of Fura-2 AM-loaded cells was measured by using continuous rapid alternating excitation from monochromators (340 and 380 nm) and emission at 510 nm in a fluorescence spectrophotometer equipped with a xenon lamp (PTI, NJ, U.S.A.). The fluorescence ratio was recorded every 0.1 s using Felix Fluorescence Analysis Software.

**Chemotaxis.** Dami cells were cultured one night in medium without serum at the density of 0.25 x 10^6^ cells/ml. Migration was assessed using 8 µm pore size Falcon transwells with polycarbonate membrane inserts. 10^5^ cells were loaded onto transwell inserts in medium containing RPMI / 0.1% BSA and SDF-1 diluted in RPMI was placed in the lower transwell chamber. After incubation at 37°C in a humidified atmosphere with 5% CO2, the transwell inserts were removed and migrated cells were quantified using CellTiter-Glo luminescent substrate to measure ATP, according to the protocol provided by the manufacturer (Promega, Madison, WI, USA). The level of luminescence was determined using a Luminoskan Ascent Microplate Luminometer (Thermo Fisher Scientific Inc., Waltham, MA, USA).

**Supplemental Results**

**Behavioral characterization of RGS18-/- mice.** RGS18-/- male mice and their WTs were submitted to a behavioral test battery designed to investigate a wide range of nervous system functions or their pathologies including basic sensory-motor abilities, circadian activity and food/water intake, emotional behaviors, sensory-motor gating, pain sensitivity, and susceptibility to epileptic seizures as detailed in Table S4.

RGS18-/- mice had normal body weight, body temperature and normal physical appearance (data not shown). Gross neurological examination of animals did not show any alteration of sensory or vestibular reflexes. RGS18-/- mice also showed a normal reactivity to handling and a normal exploratory activity when transferred into a novel cage.

Results from the optomotor test showed that the number of head trackings was comparable between WT and RGS18-/- mice either during photopic or during scotopic conditions, confirming thus that their visual abilities were not affected by the RGS18 mutation (data not shown).

Motor abilities were also spared in RGS18-/- mice as measured in the rotarod, grip and string tests (data not shown).

When analyzed for pain sensitivity, RGS18-/- mice had normal pain sensitivity in the tail flick test and comparable first reaction latency to WT mice in the hot plate test. On the other hand, the latency to jump out of the hotplate was significantly reduced in RGS18-/- mice as compared to the WT mice, which might be interpreted as increased central pain sensitivity (Table S5).

When placed into circadian activity cages, the spontaneous locomotor activity was comparable between WT and RGS18-/- mice (data not shown). RGS18-/- mice displayed reduced number of rears and the difference between genotypes failed short of statistical significance (data not shown). Moreover, the number of rears during habituation period was even significantly lower in RGS18-/- mice than in WT mice, mainly during the initial hours of exploration, while food and water intake were not affected by RGS18 deletion as measured during 32 hours (data not shown).

In order to evaluate the consequence of the mutation on the emotional behaviors, animals were tested in different experimental paradigms known to trigger distinctive types of stress and anxiety. In the open field test, the distance travelled was slightly reduced in RGS18-/- mice, but the difference between genotypes was not statistically significant (Figure S3A). The number of rears and the percentage of time spent in the center were comparable between the two genotypes (Figure S3B and S3C, respectively), suggesting that RGS18 mutation does not affect anxiety-related behavior in this assay. In the tail suspension test, duration of immobility and the latency to the first immobilization were comparable between the two genotypes (data not shown), suggesting that despair behavior was not affected by the RGS18 deletion.

The magnitude of the startle response to sudden high acoustic stimulus (110 dB) and to the higher pre-pulse intensity used (90dB) was significantly higher in RGS18-/- mice than in WT mice (p < 0.05) (Figure S2A). This increased startle in the RGS18-/- mice does not seem to be secondary to changes in the processing of auditory stimuli, as they displayed normal PPI levels at all pre-pulse intensities tested (70 to 90-dB, Figure S2B).

Finally, when PTZ was injected intraperitoneally at the dose of 50 mg/kg, no difference in susceptibility to seizures was observed between control and RGS18-/- mice; the number of mice showing myoclonic, clonic or tonic seizures was statistically comparable between genotypes. Clonico-tonic seizure latency also did not differ between WT and RGS18-/- mice (data not shown).

In summary, from a behavioral point of view, RGS18-/- males displayed increased startle response and reduced number of rears in the circadian activity test. These effects might be interpreted as increased reactivity/anxiety in RGS18-/- mice. However, data from the open field test did not show any significant difference between WT and RGS18-/- mice, even if RGS18-/- mice tended to be less active at the beginning of the test. RGS18 deletion also induced a subtle increase in pain sensitivity as revealed by reduced latency to jump out of the hot plate in RGS18-/- mice. Finally, basic sensory-motor abilities, food/water intake, and susceptibility to PTZ were not altered in RGS18-/- male mice.

**Supplemental References**

1. Brown SD, Chambon P, Hrabé de Angelis M (2005) Eumorphia Consortium. EMPReSS: standardized phenotype screens for functional annotation of the mouse genome. Nat Genet 37: 1155.
2. Jellali A, Meziane H, Ouagazzal AM, Rousseau S, Romand R, et al. (2005) The optomotor response: a robust first-line visual screening method for mice. Vision Res 45: 1439–1446.
